# Spontaneous Tumor Lysis Syndrome Secondary to Metastatic Small Cell Lung Cancer

**DOI:** 10.7759/cureus.34557

**Published:** 2023-02-02

**Authors:** Simran Koura, Varun Parekh, Amish D Parikh, Kiranveer Kaur, Bryan K Dunn

**Affiliations:** 1 Internal Medicine, East Carolina University Brody School of Medicine, Greenville, USA; 2 Hematology and Oncology, East Carolina University Brody School of Medicine, Greenville, USA; 3 Pulmonary and Critical Care, East Carolina University Brody School of Medicine, Greenville, USA

**Keywords:** cairo and bishop classification, metabolic acidosis, spontaneous tumor lysis syndrome, small cell lung carcinoma, tumor lysis syndrome

## Abstract

Tumor lysis syndrome (TLS) is an oncology emergency caused by the lysis of tumor cells that releases cell contents into the blood. TLS is typically associated with leukemia following the initiation of chemotherapy. Spontaneous TLS has been seen in hematologic malignancies, but the incidence of spontaneous TLS in solid tumors is rare, and only nine cases have been reported in small cell lung carcinoma. We present a case of a patient who presented with severe metabolic acidosis and electrolyte abnormalities consistent with TLS. At presentation, our patient was found to have small cell lung carcinoma with metastasis to the liver. This patient was managed with bicarbonate, rasburicase, allopurinol, and calcium replacement and started on continuous renal replacement therapy, but unfortunately was transitioned to comfort care and passed away. Risk factors for spontaneous TLS include bulky disease, elevated lactate dehydrogenase, elevated white blood cell counts, renal compromise, and abdominal organ involvement. The most common laboratory findings for TLS include metabolic acidosis and hyperuricemia, hyperphosphatemia, hyperkalemia, and hypocalcemia. Cases of spontaneous TLS, however, have been noted to have smaller elevations in phosphate levels. Spontaneous TLS is a rare but potentially fatal complication that can be seen in small cell lung carcinoma.

## Introduction

Tumor lysis syndrome (TLS) is an oncologic emergency in which a rupture of tumor cells results in a release of cellular contents into the serum, causing a metabolic acidosis accompanied by hyperuricemia, hyperphosphatemia, hyperkalemia, and hypocalcemia [1]. TLS is most often seen in leukemia and non-Hodgkin's lymphoma, with an incidence of 4-42% of hematologic malignancies, typically following cytotoxic treatment [2]. Spontaneous TLS occurs in the absence of chemotherapy and has primarily been seen in hematologic malignancies. It is rare in solid tumors and only nine cases have been reported in small cell lung carcinoma [3]. Other risk factors associated with spontaneous TLS include solid tumors with high proliferation rates and high tumor burden [4].

## Case presentation

**Figure 1:**
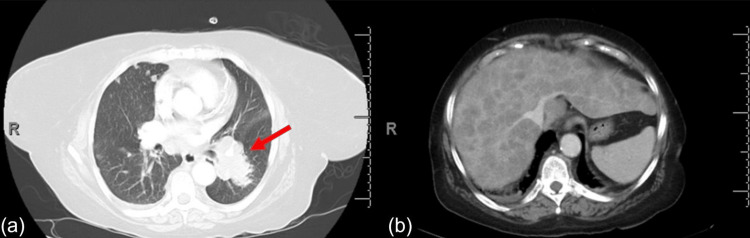
CT of the chest and abdomen demonstrating primary lesion small cell lung carcinoma (a) with metastatic liver lesions (b).

A 67-year-old female with a past medical history of hypertension, hyperlipidemia, and ethanol abuse and a 40-pack-year smoking history presented with shortness of breath, generalized weakness, and poor appetite for one week. She was found to have elevated liver function tests with aspartate aminotransferase (AST) at 728 U/L, alanine transaminase (ALT) at 315 U/L, a leukocytosis of 11 x 109/L, and a lactic acidosis of 7.5 U/L at presentation. CT of the chest, abdomen, and pelvis revealed bulky mediastinal lymphadenopathy, multiple pulmonary nodules, and several hepatic nodules suggestive of metastatic cancer of unknown origin (Figure 1). The patient was admitted to the hematology-oncology unit for further workup of malignancy.

On day five of admission, the patient underwent a liver biopsy of a metastatic lesion, which later showed poorly differentiated carcinoma consistent with small cell carcinoma of lung origin with a Ki-67 proliferative activity of 91-100%. On day six, the patient became encephalopathic with agonal respirations and was intubated and transferred to the medical intensive care unit (MICU).

At the presentation to the MICU, the patient was in vasopressor-dependent shock of unknown etiology and required inotropic support with norepinephrine and vasopressin to maintain a mean arterial pressure of 65 mmHg. Bacterial cultures were collected, and the patient was started on empiric antibiotics with vancomycin, cefepime, and piperacillin-tazobactam for concern of sepsis as the cause of the patient's vasopressor-dependent shock. Over the course of 24 hours, the patient’s labs peaked at uric acid at 13.8 mg/dL, phosphate at 7.3 mg/dL, potassium at 5.9 mEq/L, lactic acid >24 U/L, lactate dehydrogenase (LDH) >10,008 mmol/L, and serum creatinine at 2.81 mg/dL with minimum calcium of 8.5 mg/dL. The patient had a severe anion gap metabolic acidosis with a pH of 6.99 and low bicarbonate down to 5 mEq/L. These lab abnormalities raised suspicion for TLS. The patient was given bicarbonate for severe anion gap metabolic acidosis, rasburicase, and allopurinol. The patient was also started on continuous renal replacement therapy for further management of her electrolyte abnormalities. Despite these efforts, the patient was transitioned to comfort care one day after the presentation to the MICU and passed away.

## Discussion

The Cairo-Bishop classification has been used to define TLS [5]. According to the laboratory criteria, TLS is defined as two or more of the serum value changes seen in Table 1 within three days before or seven days after the initiation of chemotherapy. Clinical TLS is defined as meeting the criteria for laboratory TLS and one or more of the criteria outlined in Table 2. No diagnostic criteria have been outlined for spontaneous TLS due to the rarity of cases.

**Table 1:** According to the Cairo-Bishop classification, laboratory criteria for tumor lysis syndrome require two or more of the above lab values. Adapted from [5].

Lab	Laboratory criteria
Uric acid	≥8 mg/dL or 25% increase from baseline
Potassium	≥6 mEq/L or 25% increase from baseline
Phosphorus	≥4.5 mg/dL (adults) or 25% increase from baseline
Calcium	≤7 mg/dL or 25% decrease from baseline

**Table 2:** According to the Cairo-Bishop classification, clinical criteria for tumor lysis syndrome require meeting the laboratory criteria, in addition to at least one of the above criteria. Adapted from [5].

Clinical criteria
Creatinine ≥1.5 times the upper limit of normal
Cardiac arrhythmia or sudden death
Seizure

TLS is most often seen in patients undergoing chemotherapy or with hematologic malignancies [2]. Mortality rates from TLS in solid tumors have been reported up to 40%, being higher than the mortality rate in TLS in hematologic malignancies of 27% [1,6]. In reviewing our patient and previously reported cases, there is a 70% mortality rate in patients who developed spontaneous TLS in small cell lung carcinoma [1,3,7-12]. Identified risk factors for spontaneous TLS include solid tumors with high proliferative rates, bulky disease measuring greater than 10 cm in diameter, elevated LDH greater than two times the upper limit of normal, elevated white blood cell (WBC) count greater than 25,000/µL, pre-existing renal compromise, pre-existing uremia, dehydration, and abdominal organ disease involvement [4]. Our patient had the following risk factors: solid tumor with high proliferative rates, bulky disease, elevated LDH, elevated WBC count, and abdominal organ disease involvement.

The first case of spontaneous TLS in small cell lung cancer was reported in 2008 [1], and only nine cases have been reported to date (Table 3). One significant difference that has been noted between spontaneous TLS and cytotoxicity-induced TLS is the degree of hyperphosphatemia. Spontaneous TLS has been noted to have smaller elevations in phosphate levels. This is thought to result from the reuse of phosphate in the rapid generation of new tumor cells since spontaneous TLS is associated with malignancies with high proliferation rates [1]. Only one patient out of the nine previously reported cases of spontaneous TLS in small cell lung carcinoma presented with a phosphate level of less than 4.5 mg/dL.

**Table 3:** Summary of lab values, presence of metastasis at presentation, and mortality in the nine reported cases of spontaneous tumor lysis syndrome in small cell lung carcinoma.

Authors	Creatinine (mg/dL)	Uric acid (mg/dL)	Phosphate (mg/dL)	Potassium (mEq/L)	Calcium (mg/dL)	Survival	Presence of metastasis
Dean et al. [1]	4.1	21.7	7.1	6.2	1.07	No	Liver
Huet et al. [7]	2.29	16.5	5.1	5.9	9.5	No	Liver, bone marrow
Alan et al. [8]	2.15	20.32	5.2	6	10.2	No	Liver
Dhakal et al. [9]	2.4	17.6	7.1	7.8	10.82	No	Liver
Kanchustambham et al. [10]	0.8	8.3	5.3	6.1	Not available	Yes	None
Weerasinghe et al. [11]	3.92	16.5	4.3	7.4	8.2	Yes	Liver, vertebral body
Boonpheng et al. [12]	0.5-7.9	11.3	8.4	5.6	Not available	Yes	Liver
Martínez-Sáez et al., patient 1 [3]	0.99-3.03	15-22.6	6.2-10.7	4.6-6.3	8.6-10.4	No	Liver
Martínez-Sáez et al., patient 2 [3]	2.4-4.6	12.6-16.9	5.1-6.7	5.6-6	8.5-9	No	Liver

A common presentation for spontaneous TLS is similar to the presentation of TLS with metabolic acidosis, elevated creatinine, hyperuricemia, hyperkalemia, and hypocalcemia. The major difference from TLS is that spontaneous TLS may present with a normal or less significantly elevated phosphate level. Management of spontaneous TLS involves the correction of pH and electrolyte abnormalities. Glucose with insulin, beta-agonists, and oral potassium-binding agents can be used to correct hyperkalemia. Hyperphosphatemia should be corrected prior to calcium supplementation to avoid calcium phosphate precipitation. Restriction of phosphate intake and use of phosphate-binding agents can correct hyperphosphatemia. Allopurinol is useful in the prophylaxis of TLS; however, rasburicase should be used to break down already-formed uric acid crystals. Additionally, continuous renal replacement therapy can further correct severe electrolyte abnormalities [8].

## Conclusions

Spontaneous TLS is a rare and potentially fatal complication that healthcare providers should bear in mind in aggressive cancers such as small cell lung carcinoma. Spontaneous TLS seems to mostly occur in small cell lung carcinoma patients who present late with evidence of high-burden metastatic disease. Risk factors that make spontaneous TLS more likely include bulky disease and tumors with high proliferative rates. It presents similar to classic cases of TLS; however, phosphate levels may not be as elevated.
